# Analysis of the First Temperate Broad Host Range Brucellaphage (BiPBO1) Isolated from *B. inopinata*

**DOI:** 10.3389/fmicb.2016.00024

**Published:** 2016-01-28

**Authors:** Jens A. Hammerl, Cornelia Göllner, Sascha Al Dahouk, Karsten Nöckler, Jochen Reetz, Stefan Hertwig

**Affiliations:** Department of Biological Safety, Federal Institute for Risk AssessmentBerlin, Germany

**Keywords:** *Brucella*, phage, genome, temperate, lysogeny, prophage

## Abstract

*Brucella* species are important human and animal pathogens. Though, only little is known about mobile genetic elements of these highly pathogenic bacteria. To date, neither plasmids nor temperate phages have been described in brucellae. We analyzed genomic sequences of various reference and type strains and identified a number of putative prophages residing within the *Brucella* chromosomes. By induction, phage BiPBO1 was isolated from *Brucella inopinata*. BiPBO1 is a siphovirus that infects several *Brucella* species including *Brucella abortus* and *Brucella melitensis*. Integration of the phage genome occurs adjacent to a tRNA gene in chromosome 1 (chr 1). The bacterial (*attB*) and phage (*attP*) attachment sites comprise an identical sequence of 46 bp. This sequence exists in many *Brucella* and *Ochrobactrum* species. The BiPBO1 genome is composed of a 46,877 bp double-stranded DNA. Eighty-seven putative gene products were determined, of which 32 could be functionally assigned. Strongest similarities were found to a temperate phage residing in the chromosome of *Ochrobactrum anthropi* ATCC 49188 and to prophages identified in several families belonging to the order rhizobiales. The data suggest that horizontal gene transfer may occur between *Brucella* and *Ochrobactrum* and underpin the close relationship of these environmental and pathogenic bacteria.

## Introduction

Bacteriophages (phages) are viruses which exclusively infect bacteria. They have been found in nearly all known bacterial taxa and hence exist in most ecosystems (Wommack and Colwell, 2000; Prestel et al., 2008; Srinivasiah et al., 2008). Phages are either directly associated with their bacterial hosts or occurring in large numbers as free particles in the environment. The global phage population has been estimated at 10^31^ particles, ~10 times more than their host cells (Hatfull, 2008). Thus, phages are the most abundant biological entities on earth. They play a major role in horizontal gene transfer by either transduction of bacterial genes, which can be provoked by both temperate and virulent phages, or by lysogenic conversion, a change in the properties of a bacterial cell as a result of its infection with a temperate phage (Fortier and Sekulovic, 2013; Brown-Jaque et al., 2015; Penadés et al., 2015). Phage-mediated gene transfer can modulate the virulence of pathogenic bacteria. Many bacterial toxins are encoded by temperate phages, e.g., botulism toxin, cholera toxin, diphtheria toxin, or shiga toxins (Brüssow et al., 2004; Casas and Maloy, 2011). In addition, phages may encode various enzymes important for pathogenicity such as phospholipase, staphylokinase, superoxide dismutase, and effector proteins participating in adhesion, invasion, and serum resistance (Boyd and Brüssow, 2002). Prophages are common in bacterial chromosomes and can constitute as much as 10–20% of a bacterium's genome (Casjens, 2003). Strains containing multiple sequence-related prophages can experience recombinations leading to prophages with new gene constellations (Canchaya et al., 2004). Moreover, prophages can cause large-scale rearrangements of the bacterial chromosome (Iguchi et al., 2006). Though, while temperate phages, some of which encoding virulence factors, have been identified in many pathogenic species, they have yet not been reported for some intracellular pathogens like *Coxiella, Rickettsia*, and *Brucella*.

Brucellae are facultative intracellular pathogens that belong to Alphaproteobacteria. The genus *Brucella*, established in 1920 by Meyer and Shaw, consists of 11 species, which can be divided into the classical *Brucella* species (*B. melitensis, B. abortus, B. suis, B. canis, B. ovis*, and *B. neotomae*), brucellae isolated from marine mammals (*B. ceti* and *B. pinnipedialis*) and the more recently discovered species *B. microti, B. papionis*, and *B. inopinata* (http://www.bacterio.net/brucella.html). *B. inopinata*, recovered from a breast implant infection, is currently represented by a single strain (Scholz et al., 2010). All *Brucella* species are genetically highly related with genome similarities of >90% at the nucleotide level. While most *Brucella* species are fastidious and slow growing with limited metabolic activity, *B. microti* and *B. inopinata* are fast growing bacteria, with a biochemical profile similar to that of *Ochrobactrum* (Al Dahouk et al., 2010), the closest genetic neighbor within the family *Brucellaceae* that shows strong 16S rRNA and *recA* similarities to *Brucella* (e.g., *Ochrobactrum anthropi* 98.7 and 85.5%, respectively, Scholz et al., 2008). Consequently, *B. microti* and *B. inopinata* are often misidentified as *Ochrobactrum* using commercially available biochemical test systems, like e.g., API20NE.

Brucellae can cause brucellosis, a widespread bacterial zoonotic disease (Zheludkov and Tsirelson, 2013) leading to reproductive failure and abortion in animals and a feverish multiorgan disease in humans (Godfroid et al., 2012). The species mainly associated with human infections are *B. melitensis* transmitted from sheep and goats, *B. abortus* from cattle and *B. suis* from pigs. However, not only livestock but also wildlife can contribute to the spread of this pathogen and may pose a risk (Zheludkov and Tsirelson, 2013; Hammerl et al., 2015). The adaption of brucellae to the intracellular lifestyle within a specific host is known to be associated with genome reduction (Teyssier et al., 2004). Genes not necessary for survival in the intra-host environment are lost. In contrast, the exchange and acquisition of genetic material is essential for free-living bacteria in water and soil which have to struggle against changing conditions. With the discovery of various new and atypical *Brucella* species, the evolution from a soil progenitor to a highly pathogenic intracellular bacterium is broadly discussed (Wattam et al., 2014). Lateral gene transfer is supposed to be responsible for the introduction of virulence factors into *Brucella*, but underlying mechanisms have not yet been identified. Numerous genes presumably important for survival and virulence have been detected in the chromosomes of *B. abortus, B. melitensis*, and *B. suis*; some of them are located on genomic islands (Delrue et al., 2004). By contrast, neither plasmids nor temperate phages have to date been isolated from *Brucella*, even though virulent *Brucella* phages devoid of virulence-associated genes are routinely employed for typing. The history of brucellaphages and their use as diagnostic tool for the identification of *Brucella* species began with the discovery of phage Tb (Tbilisi, Russia) in the 1960s (Corbel and Phillip, 1972; Corbel and Thomas, 1976; Thomas and Corbel, 1977). Some phages were isolated from *Brucella* cultures but lysogeny has not yet been demonstrated. On the basis of their host range, brucellaphages are classified in seven groups (Tb, Fi, Wb, Bk2, R/C, Iz, Np, Alton et al., 1975; Ackermann et al., 1981; Corbel, 1987). All brucellaphages described so far have a podoviral morphology and are closely related (Flores et al., 2012; Farlow et al., 2014). They are considered as a single taxonomic species comprising different host range variants (Corbel and Thomas, 1976; Ackermann et al., 1981). Analyses of whole genome sequences of *Brucella* strains imply that temperate phages might also occur in this genus since prophage DNA has been detected in some of the investigated strains. The genome of a putative prophage of *Roseobacter* has e.g., been found in *B. canis* SVA13 (Kaden et al., 2014a,b). However, there is yet no information available on the integrity of the phage. The hitherto identified prophage sequences in *Brucella* might be remnants of formerly intact phage genomes that were lost on the way of the bacteria from soil to mammalian hosts.

The goal of this study was to ascertain, whether intact prophages exist in *Brucella*. To accomplish this, the prophage content of numerous *Brucella* reference and type strains has been determined by *in silico* analyses. Induction experiments were carried out to release phage particles from the bacteria. By doing this, the temperate phage BiPBO1 was isolated from *B. inopinata* and characterized in terms of its phenotypic and genotypic properties.

## Materials and methods

### Bacterial strains, media, and growth conditions

All strains used in this study are listed in Table S1. If not stated otherwise, *Brucella* strains were cultivated in *Brucella*-broth (Carl Roth, Karlsruhe, Germany) under microaerobic conditions (10% CO_2_) according to standard procedures. For the cultivation of *B. ovis*, the medium was supplemented with 10% horse serum (Alton et al., 1975). Solid and overlay agar contained 1.8% and 0.7% (w/v) bacto-agar No. 1 (Oxoid, Wesel, Germany), respectively.

### Prophage induction experiments

To study phage release from brucellae, several stress conditions (mitomycin C treatment, high temperatures, and UV radiation) were applied. At a McFarland of 0.8–1.2, 12.5 μg/ml^−1^ mitomycin C, heat treatment at 60, 70, or 80°C (for 30 and 60 s), or UV radiation (for 30, 60, and 120 s) were applied and cultivation of the bacteria was continued for 16 h without shaking. Induction via UV treatment was performed by pouring an aliquot of the *Brucella* culture into a sterile petri dish (*d* = 90 mm). The dish was placed in a distance of 10 cm to the UV lamp (corresponding 45 J m^−2^) followed by radiation for different time periods. All strains were investigated in triplicate under the respective induction conditions. Lytic activity caused by induced phages was determined as stated below.

### Isolation, propagation, and purification of BiPBO1

Phage BiPBO1 was recovered by mitomycin C treatment of *B. inopinata* strain BO1 (Scholz et al., 2010). Bacteria-free phage lysates were obtained by centrifugation at 6000 g for 15 min and filtration of the supernatant through 0.45 μm and 0.22 μm sterile filters (GE Healthcare, Munich, Germany). Lytic activity was detected by spotting 10 μl aliquots of a 1:10 dilution series of lysates onto lawns of *Brucella* indicator strains (Viazis et al., 2011). To determine phage titers, the softagar overlay method was applied (Sambrook and Russel, 2001). BiPB01 isolation was achieved by three-fold repetition of single plaque assays using *B. abortus* S19 as host. High-titer lysates were obtained by infecting five 100 ml cultures of *B. abortus* S19 (McFarland 3.0–4.0) with BiPBO1 at a multiplicity of infection (MOI) of 0.1. The lysates were prepared as stated above. After filtration, 10 mM MgCl_2_, 10 μg ml^−1^ DNaseI, and RNase A (Roche, Mannheim, Germany) were added to the lysates which were incubated at 37°C for 2 h. Phage particles were concentrated using Vivaspin® 20 columns as recommended by the manufacturer (Sartorius, Goettingen, Germany). Concentrated phages were purified by discontinuous gradient centrifugation (at 141,000 × g for 18 h) using caesium chloride (CsCl, 1.3–1.7 g ml^−1^) (Sambrook and Russel, 2001). Phage bands were collected from centrifugation tubes using a syringe and desalted by 100K Amicon Ultra centrifugal filter columns (Merck Millipore, Schwalbach, Germany).

### Determination of the host range

The host range of BiPBO1 and of two virulent reference phages F1 and F25 as a control (Table S2) was determined by spot activity assays (Viazis et al., 2011). F1 and F25 are routinely used in our institute for *Brucella* typing (Al Dahouk et al., 2012). Sequence analysis of F1 revealed that it is a close relative of phage Tbilisi (Hammerl et al., 2014). Two hundred microliters of each *Brucella* strain were mixed with 5 ml of pre-warmed *Brucella-*broth soft agar (0.7%) and poured onto a *Brucella* agar plate. Ten microliters aliquots of 1:10 serial dilutions of BiPBO1 lysates were spotted onto the overlay agar. Agar plates were visually inspected after incubation for 24 and 48 h at 37°C. Phage activity was analyzed on 26 *Brucella* reference and type strains as well as on 119 *Ochrobactrum*, seven *Yersinia enterocolitica* O:9, six *Mesorhizobium*, five *Sinorhizobium*, and five *Pseudomonas* strains.

### Lysogenisaton of *B. abortus* S19

The ability of BiPBO1 to lysogenize *B. abortus* S19 was investigated by isolation of bacteria from lysis zones. The respective area of the soft agar was removed and bacteria were eluted in PBS buffer. One hundred microliters aliquots of 1:10 dilution series of the bacterial suspensions were plated on *Brucella* agar and incubated for 48°h at 37°C. Altogether 50 colonies were investigated in terms of immunity against superinfection with BiPBO1. This was performed by spot assays as described above. The non-treated *B. abortus* S19 strain was used as control to demonstrate lytic activity. Colonies identified as phage resistant were further screened for phage release by induction with mitomycin C. Positive colonies were selected for further analyses. The stability of lysogeny was investigated by cultivation of the lysogens over 50–100 generations.

### Phage curing

Curing was examined by treating the lysogenic strains *B. inopinata* BO1 and *B. abortus* S19lysBiPBO1 with acridine orange (final concentration 0.1, 0.05, and 0.01 mM). At a McFarland of 0.8–1.2, acridine orange was applied and cultivation was continued for 16 h without shaking. Thereafter, 1:10 dilution series of the treated cultures were plated on *Brucella* agar and cultivated as stated above (Hammerl et al., 2008). Colonies from selected dilutions were analyzed for BiPBO1 phage activity (see above) and by Multiplex-PCR using primers deduced from the genes for the integrase (ORF37), major capsid protein (ORF06), and replication protein (ORF79, Table S5).

### Phenotypic profiling via Micronaut^TM^

Differences in the phenotypic profile (metabolization of various substrates) between *B. abortus* S19 (phage-free) and *B. abortus* S19lysBiPBO1 were investigated as previously described (Al Dahouk et al., 2010). Final data are based on results of three independent experiments.

### PCR analyses

PCR reactions were performed in an Eppendorf Mastercycler ep Gradient (Eppendorf, Hamburg, Germany) according to standard protocols. Single reactions were carried out in a final volume of 25 μl by use of DreamTaq DNA polymerase amplification components (Fisher Scientific, Schwerte, Germany). The mastermix of a reaction comprised 9.0 μl RNase-free water, 2.5 μl 10 × DreamTaq buffer, 2.5 μl dNTP solution (2 mM), 6.5 μl of DreamTaq Enzyme, 2.5 μl of each primer, and 2.0 μl of template DNA (~10 ng/μl). Primers were designed by means of Accelrys Gene v2.5 (Accelrys Inc., San Diego, CA, USA) using default parameters. For the detection of prophage DNA by Multiplex-PCR, reactions started with activation and template denaturation at 94°C for 120 s followed by 35 cycles (denaturation at 94°C for 15 s, annealing at 58°C for 15 s, and elongation for 60 s at 72°C). In addition, a final elongation step at 72°C for 1 min was performed before the PCR reactions were stored at 4°C until further processing. For the determination of the chromosomal integration site, the annealing temperature and the elongation time were modified to 55°C and 120 s, respectively.

### Determination of the integration site

To determine the chromosomal integration site of the phage, gDNA of *B. inopinata* BO1 and *B. abortus* S19lysBiPBO1 was isolated using the DNA mini preparation kit (Qiagen, Hilden, Germany). The DNAs were digested with several restriction endonucleases (Bsp143I, DpnI, DraI, Eco32I, HindIII) according to the manufacturer's recommendations (Fermentas, St. Leon Roth, Germany). Restriction fragments of each digest were treated with T4 ligase (Fermentas) and used as template in a PCR reaction applying outward facing primers deduced from the coding region of the BiPBO1 integrase gene (Table S5). PCR products were purified (QIAquick PCR purification kit, Qiagen) and sequenced. The obtained nucleotide sequences of the amplicons were compared with whole genome sequences of the respective strains. To study the BiPB01 integration site in other *Brucella* strains, a Multiplex-PCR was developed. Primers deduced from the *Brucella* chromosome left and right of the BiPB01 integration site provided information on the presence of foreign DNA at this position. Combinations of these primers with primers deduced from the BiPB01 genome were used to detect BiPB01-related prophage DNA. All primers are listed in Supplementary Material Table S5.

### SDS-PAGE and mass spectrometrical analysis of structural proteins

The composition of structural proteins of CsCl-purified phage particles was investigated by Coomassie stained SDS-PAGE according to standard procedures. Protein bands were excised and prepared for tryptic *in gel*-digests as previously described (Hammerl et al., 2011). The prepared samples were investigated by MALDI-TOF-TOF MS/MS analysis. Mass spectra were interpreted using the Mascot software and NCBI database.

### Transmission electron microscopy

CsCl-purified phages were applied to pioloform-carbon-coated, 400-mesh copper grids (Plano GmbH, Germany), for 7 min, fixed with 2.5% aqueous glutaraldehyde solution for 1 min, stained with 2% aqueous uranyl acetate solution for 1 min and examined by transmission electron microscopy using a JEM-1010 (JOEL, Japan) at 80 kV accelerated voltage.

### Extraction of phage DNA and sequencing

Phage DNA was extracted from CsCl-purified particles by proteinaseK/SDS treatment followed by phenol/chloroform extraction and ethanol precipitation (Sambrook and Russel, 2001). Thereafter, the phage DNA was resuspended in 0.5 × TE-buffer (pH 8.0) for further analyses.

Whole-genome sequencing was performed with the Roche 454 genome sequencer FLX titanium system by LGC Genomics (Berlin, Germany). Library generation for 454 FLX sequencing was carried out according to the manufacturer's standard protocols (Roche/454 Life Sciences, Branford, Connecticut, USA). Phage DNA was sheared by nebulization into fragments between 500 and 1000 bp. Fragments were end-polished and the required adaptors for sequencing were ligated to the ends of the fragments. Sequencing was performed on 1/16 picotiterplate (PTP) on the GS FLX using Roche/454 Titanium chemistry. Sequence reads were assembled using the Roche/454 Newbler software at default settings (454 Life Sciences Corporation, Software release 2.3) resulting in one contig with an average sequence coverage of more than 90 per consensus base.

### Bioinformatic analysis

To identify putative prophage sequences in the available *Brucella* spp. genomes of GenBank (NCBI), the Phage Search Tool-PHAST was used (Zhou et al., 2011). Sequence analysis and alignments were carried out using Accelrys Gene v2.5 (Accelrys Inc., San Diego, CA, USA). ORF analyses were performed using the algorithms of MyRAST (Aziz et al., 2008; Meyer et al., 2008; Glass et al., 2010) and ORF Finder (NCBI) (gene product size >29 aa, Rombel et al., 2002). Similarity and identity values were determined at the NCBI homepage using the standard parameters of different BLAST algorithms (Johnson et al., 2008). Transcription terminators were identified using TransTerm (Brown et al., 1993; Ermolaeva et al., 2000) and Arnold (Naville et al., 2011). If not stated otherwise, standard parameter of the respective algorithms were used.

### Nucleotide sequence accession number

The complete nucleotide sequence of the BiPBO1 genome was submitted to GenBank under the accession number KT724718.

## Results

### Prophages are common in *Brucella*

In this *in silico* study we wanted to gather information about the occurrence of prophage sequences within the genus *Brucella*. Using the PHAST computer program, various sequenced *Brucella* reference and type strains (Table 1) were analyzed. Except for the *B. inopinata* strain BO1, which has only partially be sequenced (WGS), all studied *Brucella* strains exhibited putative prophage DNA. Up to four individual prophage regions between 7.7 and 83.1 kb in size were identified within each genome (Table 1). Highly prevalent was a very similar 9.7–13.7 kb region that we found in many *Brucella* species. This DNA region revealed strong homologies to the virulent phage P12026 of the marine bacterium *Marinomonas* (Kang et al., 2012). Next frequently, a DNA region of 9.5–83.1 kb was detected in six *Brucella* species showing relationship to a prophage already identified in the *B. suis* strain 1330 (Paulsen et al., 2002). In contrast to these prophage DNAs existing in several *Brucella* species, only the three investigated *B. melitensis* strains contained a putative prophage related to the *Trichoplusia ni* ascovirus 2c that causes chronic disease in lepidopteran larvae (Wang et al., 2006) Prophage DNA similar to the virulent *Roseobacter denitrificans* phage RDJLPhi1 (Zhang and Jiao, 2009) was exclusively detected in *B. ceti*, while *B. abortus* 544 contains prophage sequences similar to the giant moumouvirus isolated from the protozoa *Acanthamoeba polyphaga* (Yoosuf et al., 2012). Finally, four prophage DNA regions related to phages or prophages of *Marinomonas, Pseudomonas, Rhodobacter*, and *E. coli* were found in the *B. inopinata*-like strain BO2.

**Table 1:** **Prophage sequences in *Brucella* determined by *in silico* analysis (PHAST)**.

***Brucella* strains**	**Bio-var (bv)**	**Acc. No. (NCBI)**	**Related phages (Organism)**	**Size (kb)**	**Status (PHAST)**	**Score**	**CDS**	**GC content (%)**
***B. abortus***								
544	bv 1	JPHK01000000^*^	Moumouvirus (*Acanthamoeba*)	17.5	Incomplete	10	14	58.0
			P12026 (*Marinomonas*)	13.7	Questionable	90	18	61.2
86/8/59	bv 2	ACBJ00000000^*^	P12026 (*Marinomonas*)	13.6	Intact	130	17	61.2
Tulya	bv 3	ACBI00000000^*^	P12026 (*Marinomonas*)	13.7	Questionable	80	16	61.2
292	bv 4	ACBH00000000^*^	P12026 (*Marinomonas*)	13.7	Intact	130	19	61.2
B3196	bv 5	ACXC00000000^*^	P12026 (*Marinomonas*)	13.6	Intact	110	17	61.2
870	bv 6	ACBG00000000^*^	P12026 (*Marinomonas*)	13.7	Intact	110	17	61.2
63/75	bv 7	NZ_CP007660	P12026 (*Marinomonas*)	13.7	Questionable	90	17	61.2
C68	bv 9	ACEL00000000^*^	P12026 (*Marinomonas*)	13.7	Intact	110	17	61.2
S19		NC_010742.1	Prophage (*B. suis* 1330)	31.7	Intact	150	27	52.6
***B. melitensis***								
16M	bv 1	NC_003317.1^†^	Tricho_2c	17.4	Incomplete	10	15	58.0
			Prophage (*B. suis* 1330)	22.6	Intact	150	33	51.5
63/9	bv 2	ACEM00000000.1^*^	Tricho_2c	25.2	Incomplete	20	14	56.9
			P12026 (*Marinomonas*)	13.7	Questionable	90	16	61.2
			Prophage (*B. suis* 1330)	22.1	Intact	120	22	51.3
Ether	bv 3	ACEI00000000^*^	Tricho_2c	22.6	Incomplete	10	14	58.1
			P12026 (*Marinomonas*)	13.7	Intact	110	20	61.2
***B. suis***								
1330	bv 1	NC_004310.3^†^	P12026 (*Marinomonas*)	9.7	Questionable	90	15	61.0
Thomsen	bv 2	NC_004311.2^†^	Prophage (*B. suis* 1330)	31.8	Intact	150	28	52.7
686	bv 3	ACBL00000000^*^	P12026 (*Marinomonas*)	13.7	Intact	110	19	61.2
40	bv 4	ACJK00000000^*^	P12026 (*Marinomonas*)	13.7	Intact	110	18	61.2
513	bv 5	ACBK00000000^*^	P12026 (*Marinomonas*)	13.7	Intact	110	19	61.3
***B. canis***								
RM 6/66		NC_010103.1^†^	P12026 (*Marinomonas*)	9.8	Questionable	90	16	61.0
			Prophage (*B. suis* 1330)	41.3	Intact	150	32	54.2
***B. neotomae***								
5K33		ACEH00000000^*^	P12026 (*Marinomonas*)	13.7	Questionable	90	17	61.4
			Prophage (*B. suis* 1330)	21.7	Intact	100	23	51.4
***B. ovis***								
63/290		NC_009505.1^†^	Prophage (*B. suis* 1330)	82.8	Intact	150	71	56.4
		NC_009504.1^†^	Prophage (*B. suis* 1330)	10.6	Intact	110	13	55.2
***B. ceti***								
B1/94		ACEK00000000^*^	RDJL Phi 1 (*Roseobacter*)	70.3	Intact	140	75	56.7
***B. pinnipedialis***								
B2/94		NC_015857.1^†^	P12026 (*Marinomonas*)	13.6	Questionable	90	16	61.2
			Prophage (*B. suis* 1330)	31.1	Intact	150	29	52.7
		NC_015858.1^†^	Prophage (*B. suis* 1330)	9.5	Questionable	80	9	55.1
***B. microti***								
CCM 4915 T		NC_013119.1^†^	Prophage (*B. suis* 1330)	83.1	Intact	150	79	56.5
***B. inopinata***								
BO1		ADEZ00000000.1^*^	None	–	–	–	–	–
***B. inopinata*****-like**								
BO2		ADFA00000000.1^*^	P12026 (*Marinomonas*)	13.6	Questionable	80	16	61.2
			PAJU2 (*Pseudomonas*)	20.6	Incomplete	50	7	53.5
			RcapMu (*Rhodobacter*)	7.7	Incomplete	40	8	56.9
			Prophage (*E. coli* Sakai)	17.4	Questionable	70	12	55.7

* whole genome shotgun sequence.

† complete genome.

### Isolation of the temperate phage BiPBO1

To elucidate whether the identified prophage regions represent active phages, one member of each species (*B. abortus* S19, *B. melitensis* 63/9, *B. ovis* 63/290, and the *B. inopinata*-like strain BO2) harboring various numbers of putative prophages (Table 1) was selected for induction experiments. In addition, *B. inopinata* BO1 was investigated because it is to date the only strain belonging to this species, even though it has not been completely sequenced thus far.

**Figure 1:**
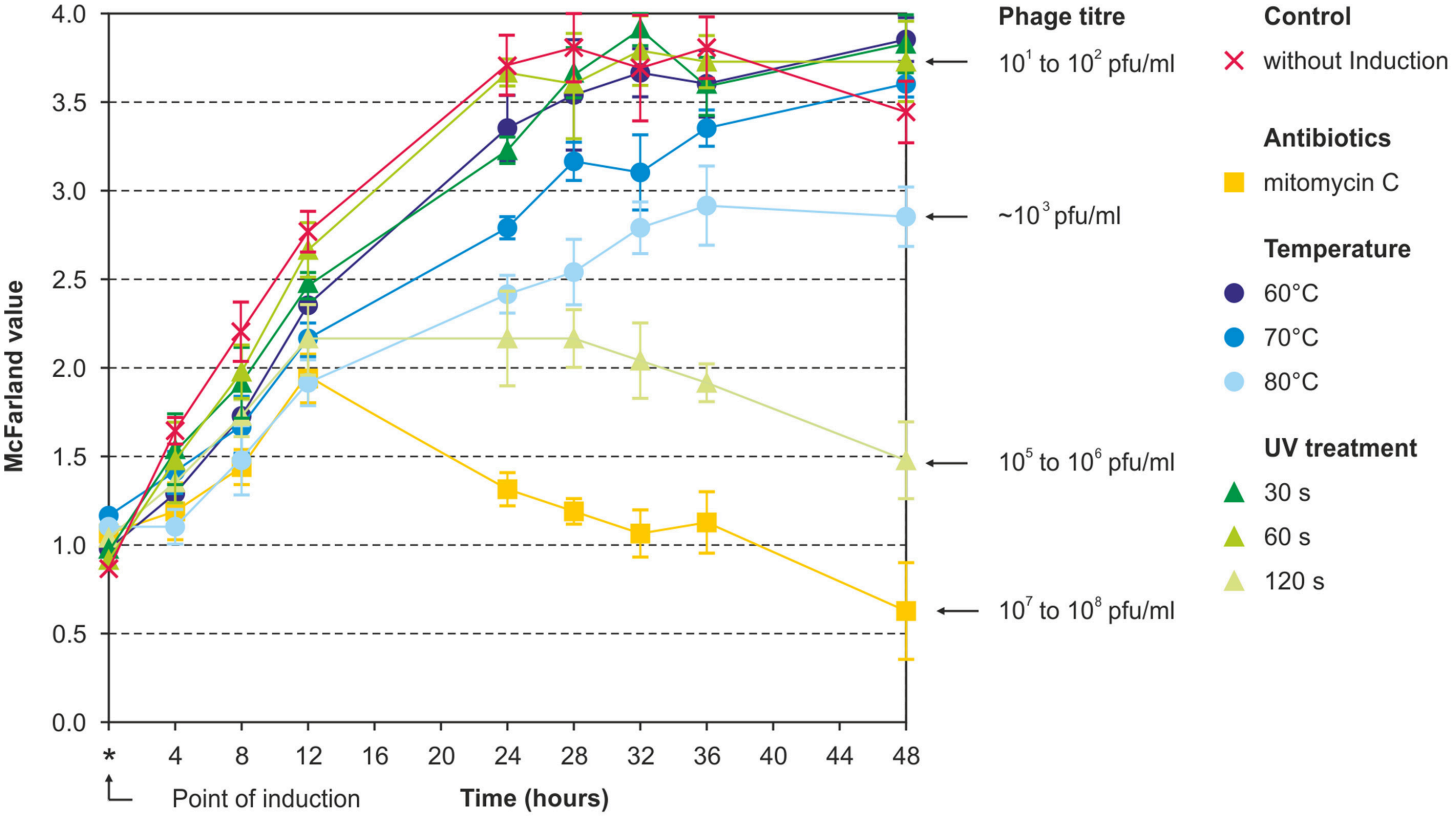
**Induction of BiPBO1 by mitomycin C, temperature, and UV**. Highest phage titers were obtained by mitomycin C, UV treatment for 120 s and heat treatment at 80°C. The diagram shows the mean value of three independent experiments. Error bars indicate standard deviations from the mean value.

**Figure 2:**
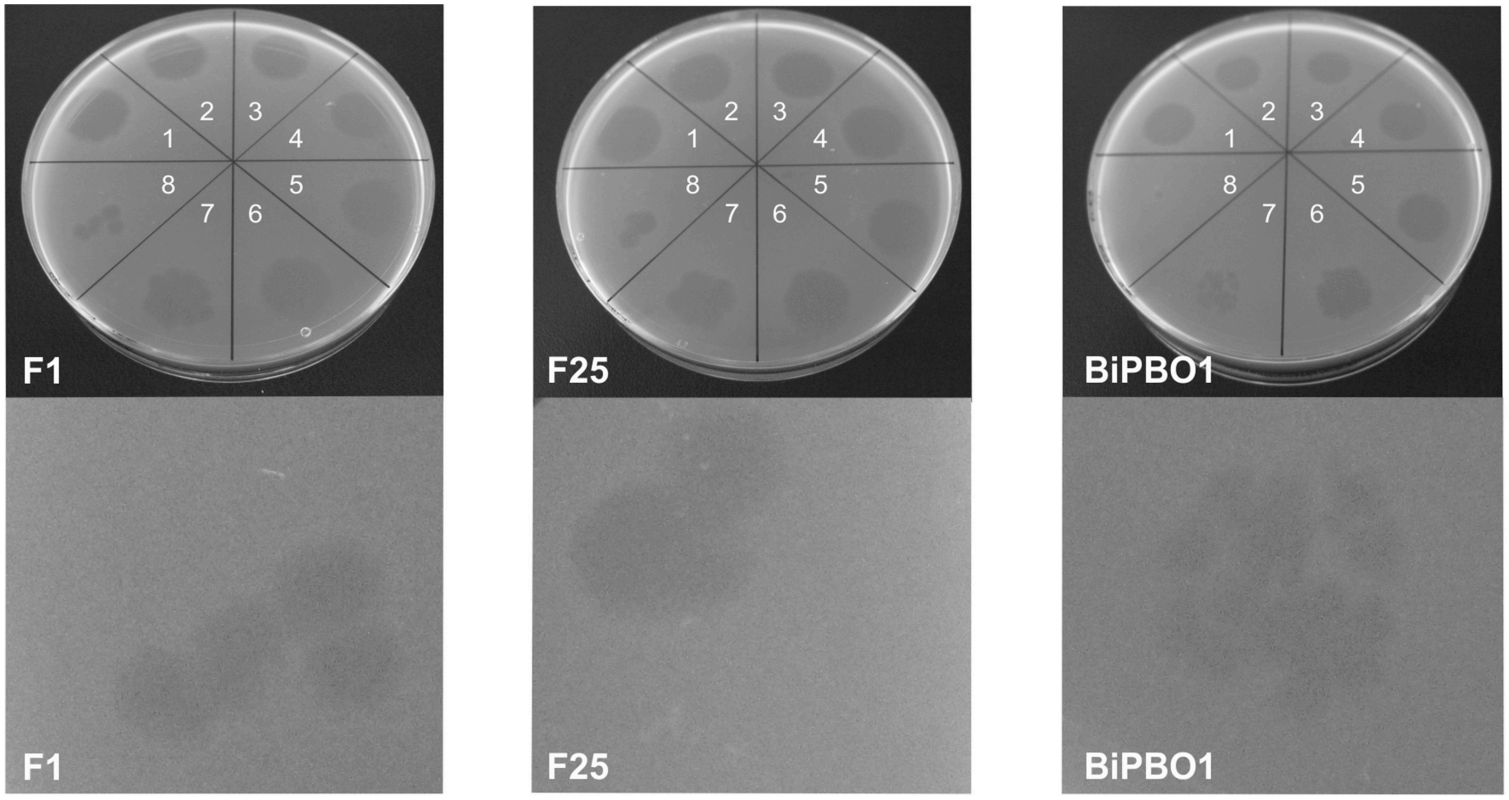
**Lytic activity of the phages BO1BiPBO1, F1, and F25**. The upper panels show spot assays (spot 1 to 8: 1:10 dilution series of the phage lysates) on *B. abortus* strain S19. The lower panels show single plaques magnified by a stereo microscope (five-fold magnification).

Prophage induction was studied by treatment of the bacteria with mitomycin C, UV and heat. Using a broad range of indicator strains belonging to several species (Table S1), no lytic activity was observed with lysates prepared from brucellae, in which prophage DNA had been detected by PHAST analysis. However, against all odds, lysates of the *B. inopinata* strain BO1, in which no prophage DNA was detected by *in silico* analysis, revealed phage-induced lysis on some *Brucella* strains. The highest phage titers were obtained using mitomycin C (5 × 10^7^ pfu/ml) while UV treatment for 60–120 s and heat treatment at 50–90°C resulted in titers of 10^5^–10^6^ pfu/ml and ~5 × 10^3^ pfu/ml, respectively (Figure 1). Plaques produced by this phage named BiPBO1 had a similar turbidity like those produced by the virulent phages F1 and F25 but were smaller in size (1–2 mm, Figure 2).

### BiPBO1 is a siphovirus with a broad host range

**Figure 3:**
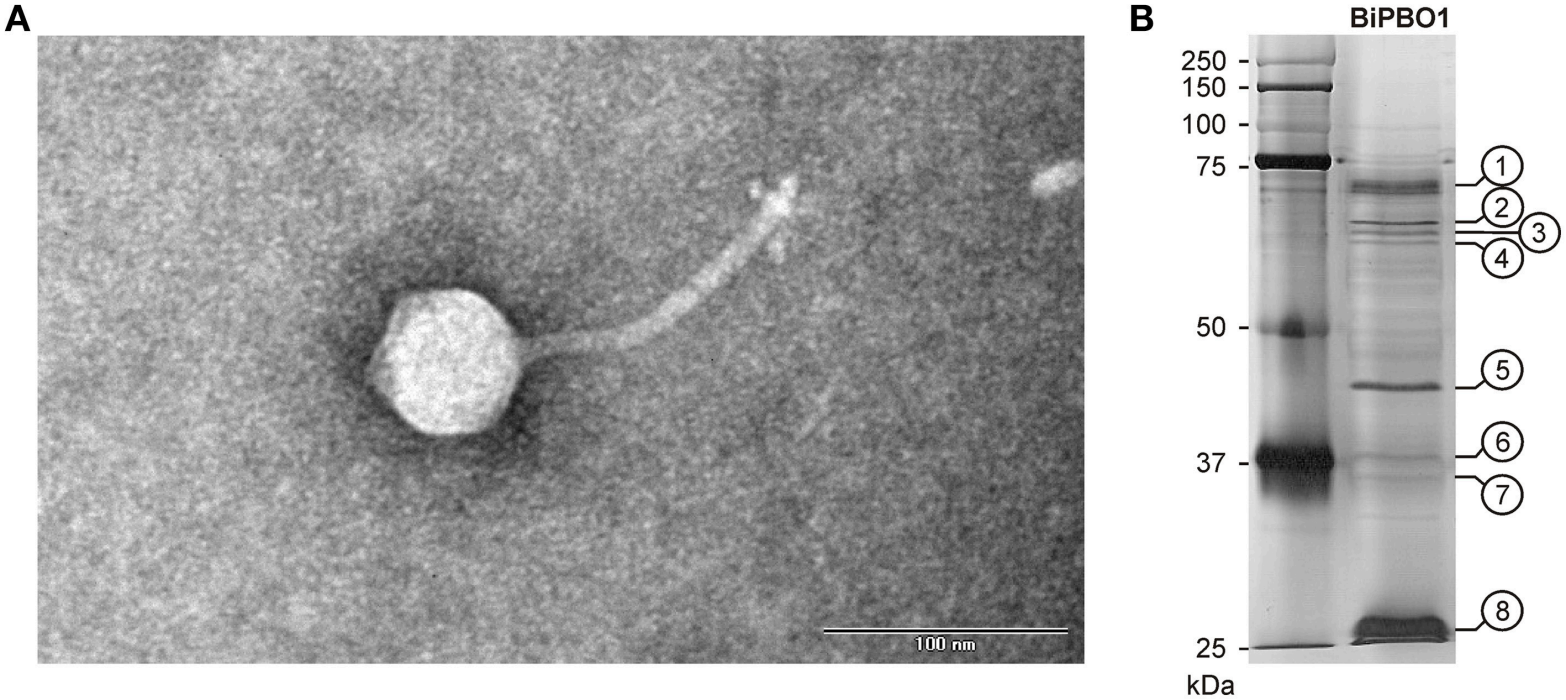
**Morphology and structural proteins of BiPBO1**. **(A)** Transmission electron micrograph (TEM) of a BiPBO1 particle isolated from *B. inopinata* strain BO1 by mitomycin C induction. The black bar represents a quantification standard of 100 nm. **(B)** SDS-PAGE profile of BiPBO1 structural proteins. Excised bands that were analyzed by mass spectrometry are numbered.

Figure 3 shows transmission electronic micrographs of phage BiPBO1. Unlike the virulent *Brucella* phages that have hitherto been described and that are all typical podoviruses (Ackermann et al., 1981), the temperate phage BiPBO1 is clearly a member of the family *Siphoviridae*. It possesses an isometric head and a long, non-contractile tail (203 × 8 nm). It is the first siphovirus isolated from *Brucella*.

The host range of BiPBO1 was determined by testing a large number of brucellae and also strains belonging to other genera. BiPBO1 lysed the *Brucella* species *B. abortus, B. melitensis, B. suis B. microti, B. pinnipedialis* and a novel *Brucella* species isolated from red foxes (Table 2), whereas strains of *Ochrobactrum* (*n* = 119), *Y. enterocolitica* O:9 (*n* = 7), *Mesorihzobium* (*n* = 6)*, Sinorhizobium* (*n* = 5), and *Pseudomonas* (*n* = 5) were not infected (data not shown). Since the phage's host specificity diverges from those of the virulent *Brucella* reference phages F1 and F25 (Table 2), BiPBO1 may be useful for typing. Similar to F1 and F25, BiPBO1 provoked growth inhibition on some indicator strains which was not associated with replication of the phage but rather caused by lysis from without (Abedon, 2011).

**Table 2:** **Host range of BiPBO1**.

***Brucella* strains**	**Biovar (bv)**	**F1**	**F25**	**BiPBO1**
***B. abortus***		**+**	**+**	**+**
S19		+	+	+
544	bv 1	+	+	+
86/8/59	bv 2	+	+	+
Tulya	bv 3	+	+	+
292	bv 4	+	+	+
B3196	bv 5	+	+	+
870	bv 6	+	+	+
63/75	bv 7	+	+	+
C68	bv 9	+	+	+
***B. melitensis***		**−**	**−**	**±**
16M	bv 1	−	−	+
63/9	bv 2	−	−	GI
Ether	bv 3	−	−	+
***B. suis***		**±**	**±**	**±**
1330	bv 1	+	+	+
Thomsen	bv 2	GI	GI	−
686	bv 3	−	−	−
40	bv 4	+	+	+
513	bv 5	+	GI	+
***B. canis***		**−**	**−**	**−**
RM 6/66		−	−	−
***B. neotomae***		**+**	**+**	**−**
5K33		+	+	GI
***B. ovis***		**−**	**−**	**−**
63/290		−	−	−
***B. ceti***		**−**	**−**	**−**
B1/94		GI	GI	−
***B. pinnipedialis***		**−**	**−**	**+**
B2/94		GI	−	+
***B. microti***		**+**	**+**	**+**
CCM 4915 T		+	+	+
***B. inopinata***		**−**	**−**	**−**
BO1		−	GI	−
***Brucella* sp. (novel)**		**+**	**+**	**+**
F60		+	+	+
F965		+	+	+

+, lysis; −, no lysis; GI, growth inhibition (no propagation of the phage).

To study the stability of lysogeny, *B. abortus* S19 was lysogenized with phage BiPBO1. Thereafter, we tried to cure the lysogenized S19 derivative and also the BiPBO1 host strain *B. inopinata* BO1 using various concentrations of acridine orange and mitomycin C. Altogether, 350 and 75 colonies of *B. inopinata* BO1 and the lysogenized *B. abortus* S19 strain, respectively, isolated after treatment with the mutagens were tested for susceptibility to BiPBO1. None of the isolates was lysed by the phage. Moreover, the BiPBO1 prophage was detected in all isolates by Multiplex-PCR and lytic particles were released from them upon induction with mitomycin C (data not shown). These data demonstrate a strong lysogenic stability of the investigated strains.

### Genome analysis of the phage

**Figure 4:**
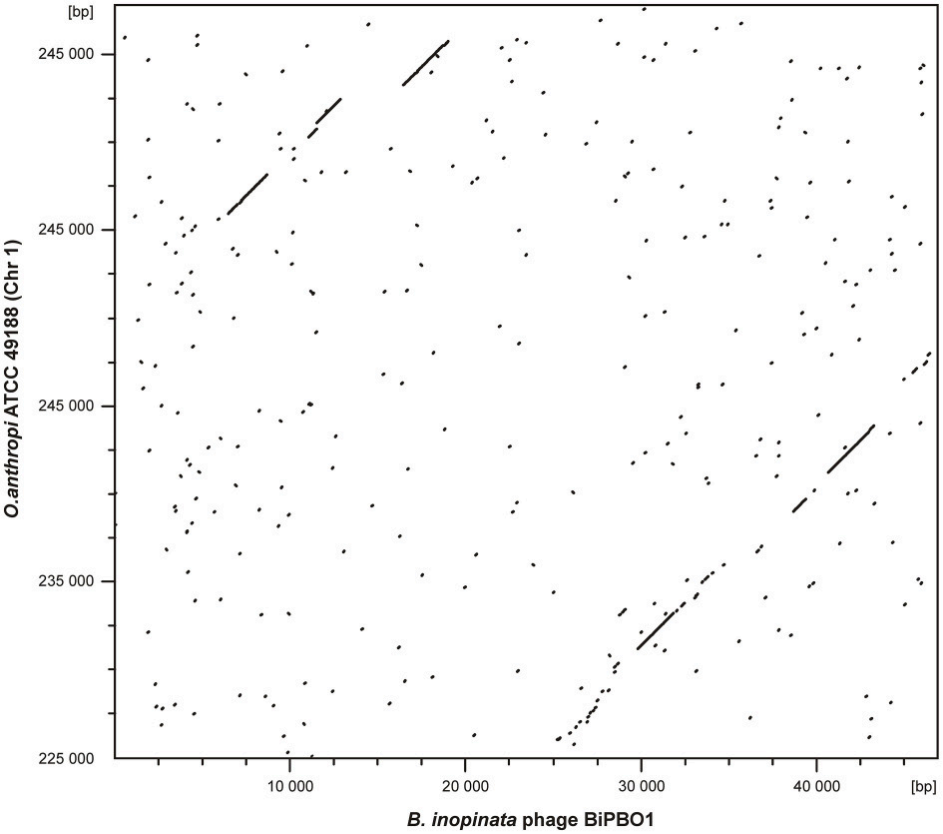
**Dot plot matrices of bacteriophage BiPBO1 with the *Ochrobactrum anthropi* ATCC 49188 chromosome 1**. The axes of abscissas and ordinates indicate the coordinates of the respective genomes. Nucleotide alignments were performed with DS Gene (version 2.5) by using 65% as a standard parameter for DNA similarity and a hash value of 6.

**Figure 5:**
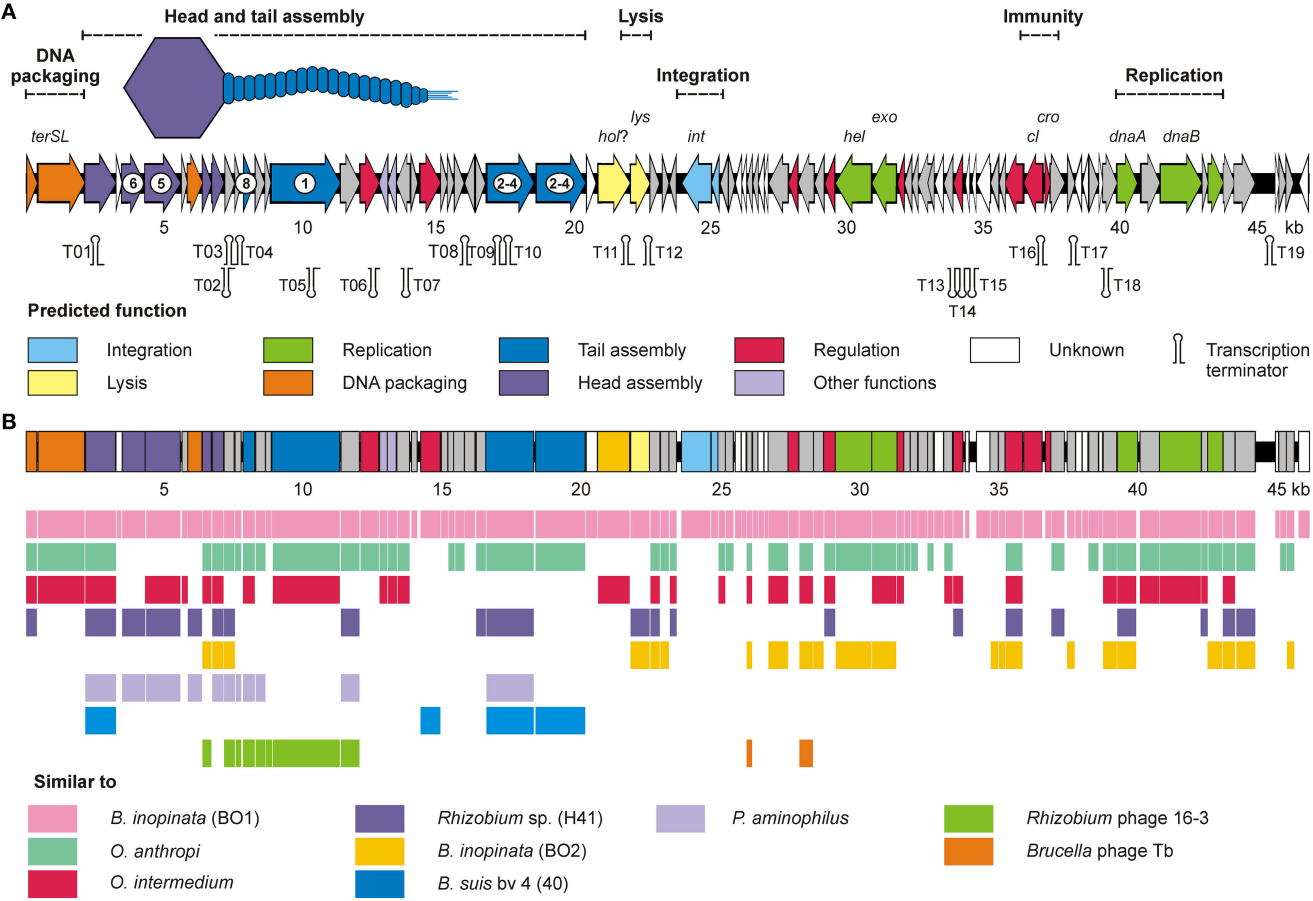
**BiPBO1 genome analysis and BO1 similarities to other prophages and phages**. **(A)** Genetic map of the BiPBO1 genome. Putative genes are colored according to the predicted functions of their products. Gene products identified by mass spectrometry are marked by encircled numbers. The position of putative Rho-independent transcription terminators (Ω) are indicated. **(B)** Synteny plot of BiPBO1 and homologies (>40% amino acid similarity) to closely related gene products of other prophages and phages.

For the isolation of BiPBO1 DNA, mass lysates originating from single plaques were concentrated and purified by CsCl-buoyant density centrifugation. Interestingly, we failed to precipitate high titres of phages by ultracentrifugation, even at high *g* values, since only tiny pellets were obtained that quickly cleared away. Therefore, particles were concentrated using Vivaspin® 20 membrane filter units. Sequencing of purified phage DNA resulted in a single contig of 46,877 bp. Bioinformatic analyses revealed 87 open reading frames (ORFs), 48 on one (plus-) strand and 39 genes on the other (minus-strand, Table S3). In addition, 19 possible transcription terminators were found, of which 11 are located on the plus-, and eight on the minus-strand (Table S4). Thirty-two of the deduced gene products could be functionally assigned, while 24 of the remaining hypothetical products did not match with any other gene sequence deposited in the NCBI database. An alignment with available sequences of *B. inopinata* BO1 revealed that only partial sequences of the BiPBO1 genome are covered by contigs of its host strain. Highest similarities were found to a putative prophage in chromosome 1 of *O. anthropi* strain ATCC 49188 (Figure 4). At the nucleotide level, 26% of the BiPBO1 genome are more than 90% identical to this prophage. Lower identity values were determined to other prophage proteins of *Brucella, Mesorhizobium, Ochrobactrum*, and *Sinorhizobium* and to some phage proteins as well (Table S3). The obtained data enabled us to construct a BiPBO1 gene map (Figure 5). The map shows that, similar to many other temperate phages, genes probably encoding structural proteins mainly reside in the left half of the genome while genes for integration and host cell lysis are located in the middle and genes involved in replication and immunity at the right end.

The first two ORFs (ORF01 and 02) probably code for the small and large subunit of the terminase. Their products are similar to many terminases encoded by prophages and also to the terminases of the *Pseudomonas aeroginosa* phage D3 (Kropinski, 2000) and Enterobacteria phage SfV (Allison et al., 2002), which possess cohesive ends. Though, restriction analyses and direct sequencing of BiPBO1 DNA did not give any indication for cohesive ends (data not shown). Two of the next ORFs may encode the portal protein (ORF03) and the major capsid protein (ORF06) that are related to head proteins of HK97-like phages. Adjacent to these ORFs, four putative tail genes (ORF09, 10, 13, and 16) were identified. ORF16 whose product is similar to tape measure proteins, is the largest ORF on the genome. All predicted structural genes of the phage are located on the plus-strand. Embedded in this part of the genome are also two genes located on the minus-strand probably encoding an addiction system composed of a toxin (20) and an antidote protein (ORF19). This module might be responsible for the observed stability of the BiPBO1 prophage. Downstream of the tail genes an ORF (ORF29) for a hypothetical protein was detected, which contains a fibronectin type 3 repeat (FN3). This domain is frequently found in tail proteins of phages and may aid in the attachment of the phage to the cell surface (Fraser et al., 2006). Interestingly, almost identical ORF29 homologs exist in many *Brucella* strains including strains that are susceptible to BiPBO1. The ~1.5 kb block within the *Brucella* genome also encompasses a sequence homologous to a part of BiPB01 ORF30 encoding a pectine lyase. Such enzymes are commonly encoded by bacteria using plant materials of the digestive tract of their hosts as a carbon source (Hugouvieux-Cotte-Pattat et al., 2012). Since ORF29 and ORF 30 are located in the vicinity of *attP* and the phage integrase gene (see below), the question arises whether these sequences are involved in integration.

The middle of the phage genome harbors numerous small ORFs with unknown functions. Two ORFs in this DNA-region probably represent genes for a phage lysin (ORF33) and the integrase (ORF37). Whether the predicted ORF32 product (a membrane acyltransferase) acts as a holin, has still to be unraveled. As with many other BiPBO1 gene products, homologies were found to bacterial proteins, but not to proteins encoded by distinct phages. In the right part of the phage genome several ORFs were identified that may be important for replication. The products of ORF77 and ORF79 are similar to a DnaA initiator protein and DnaB helicase, respectively. A second helicase may be encoded by ORF52. This gene is surrounded by an endonuclease gene (ORF51) and a gene for an exonuclease (ORF53). Besides genes probably involved in replication, this part of the genome may also contain the genetic switch of the phage. The ORF68 product is similar to prophage repressors. Close to the start codon of this ORF, the ORF69 start codon is situated on the other DNA strand. The arrangement of the ORFs 68 and 69 is reminiscent of *cI* and *cro* repressor genes but the ORF68 product did not show similarities to Cro repressors. Moreover, operator sites could not be predicted by *in silico* methods. Summing up, the analysis of the BiPBO1 genome revealed a number of ORFs which are probably essential for the propagation of the phage. On the other hand, most of the remaining genes could not be functionally assigned.

### The BiPBO1 attachment site is widely distributed in *Brucella* and *Ochrobactrum*

**Figure 6:**
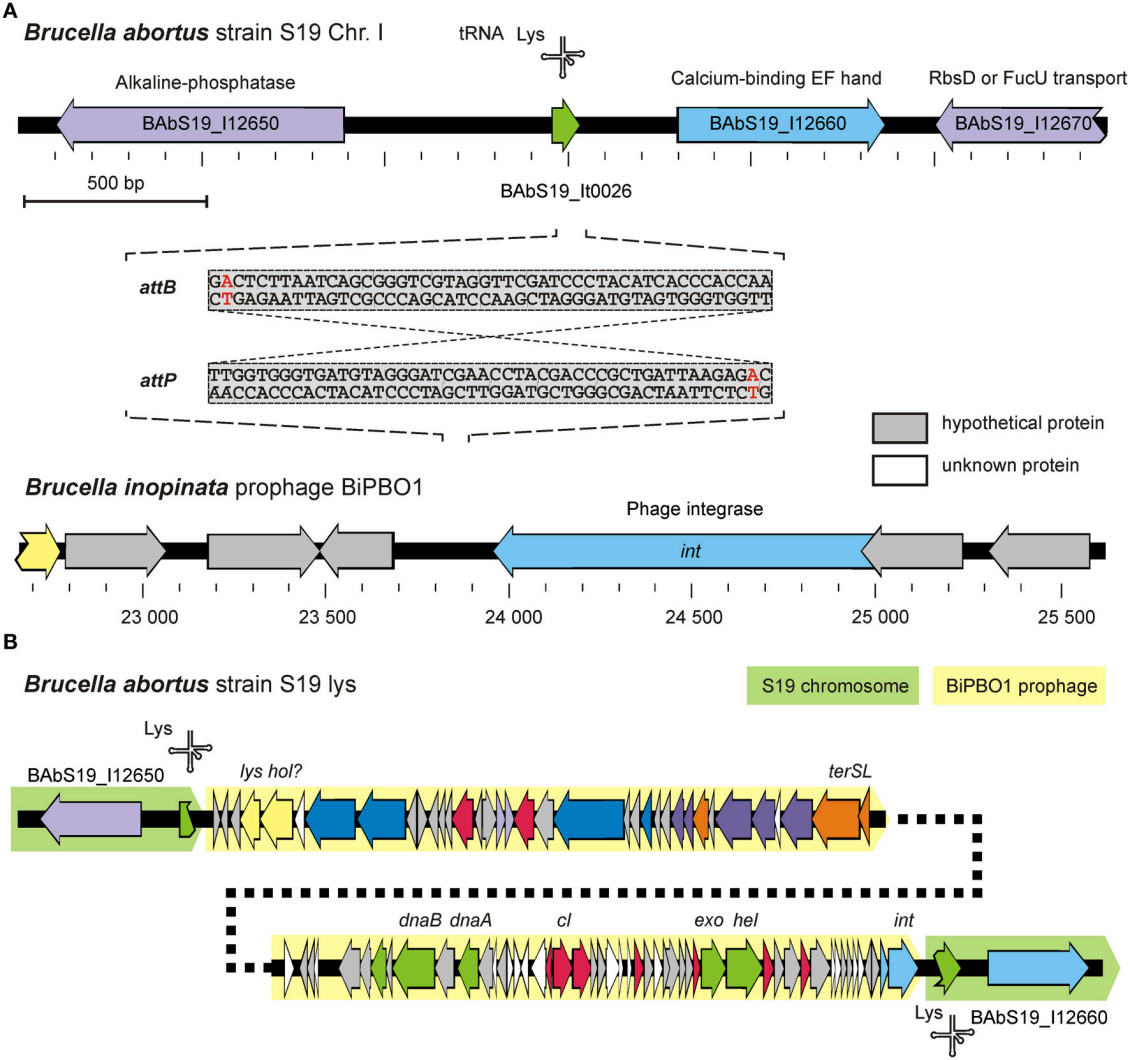
**Determination of BiPBO1 chromosomal integration. (A)** Scheme of the genomic regions of *B. abortus* S19 and BiPBO1 involved in phage integration. *attB* and *attP* sequences of S19 and BiPBO1, respectively, are given. The red nucleotide within each sequence shows the single nucleotide deviation. **(B)** Structure of the BiPBO1 prophage in the lysogenized *B. abortus* S19 strain.

In this set of experiments the BiPBO1 integration locus within the *Brucella* chromosome was characterized. In addition, we studied physiological changes that might arise through lysogenization. To determine the bacterial (*attB*) and phage (*attP*) attachment sequences, chromosomal DNA was isolated from the *B. inopinata* strain BO1, digested with several restriction endonucleases and used as template for an outward PCR with primers deduced from the BiPBO1 integrase gene. Sequencing of ~700 bp and ~1.2 kb PCR amplicons obtained by use of the restriction endonucleases DpnI and Eco32I, respectively, revealed the same transition point from the BiPBO1 prophage to the BO1 chromosome. The phage DNA was inserted adjacent to a gene for a lysine tRNA. Exactly the same integration site was found in the lysogenized *B. abortus* strain S19 (Figure 6). With the help of primers derived from the integration locus of the *Brucella* strain and the BiPBO1 genome, both borders of the integrated prophage were analyzed by sequencing of PCR products (data not shown). We found almost identical 48 bp DNA sequences in the host and the phage DNA, which probably represent the *attB* and *attP* sites, respectively. The 46 bp core sequence exists in a large number of *Brucella* and *Ochrobactrum* strains and in *Liberibacter crescens* (Leonard et al., 2012). Additionally, other members of the order rhizobiales (i.e., *Rhizobium* sp. *Neorhizobium* sp., *Agrobacterium tumefaciens, Nitrobacter* sp., *Bartonella* sp., and *Oligotropha* sp.) contain this sequence, each with a single nucleotide exchange (data not shown). A Multiplex-PCR system was developed to determine, whether this site is a hot spot for phage integration (see Section Materials and Methods). However, in none of the investigated *Brucella* reference and type strains, a prophage was detected at this locus. This does not inevitably mean that BiPBO1 related sequences do not occur in other brucellae. Therefore, the strains were also investigated for the presence of BiPBO1-related sequences (genes for the integrase, the major capsid protein, and a replication protein). Similarly to the experiment before, no PCR amplicons were obtained with the selected primers indicating that BiPBO1-like prophages are rare in *Brucella* (data not shown).

Using the Micronaut™ system (Merlin Diagnostika GmbH, Germany) phenotypic properties of *B. abortus* S19 and its lysogenic derivative were compared. We did not detect any differences between these strains. Hence, integration of BiPBO1 into the S19 chromosome does apparently not affect the metabolization of various substrates.

## Discussion

Only little is known about mobile genetic elements of *Brucella* which is an important zoonotic pathogen. Indeed, while some genomic islands have been identified in this genus (Delrue et al., 2004) and while virulent phages are routinely used for typing (Ackermann et al., 1981; Corbel, 1987; Hammerl et al., 2014), there are no reports yet on plasmids and temperate phages that may be involved in horizontal gene transfer. Even though some phages have been isolated from *Brucella* cultures (Corbel and Thomas, 1976; Los and Wegrzyn, 2012), lysogeny has not yet been demonstrated thus far. Since bioinformatic analyses of the hitherto sequenced *Brucella* phages did not reveal any prophage repressor genes (Flores et al., 2012; Farlow et al., 2014; Hammerl et al., 2014), association of the bacteria with these phages may represent a kind of pseudolysogeny (Los and Wegrzyn, 2012).

In this study, the first temperate *Brucella* phage has been characterized. Phage BiPBO1 isolated from *B. inopinata* is a siphovirus and therefore morphologically different from all known virulent *Brucella* phages, which belong to the family *Podoviridae* (Ackermann et al., 1981; Corbel, 1987; Rigby et al., 1989). Temperate phages might be more common in *Brucella* than previously expected. We detected prophage DNA in many *Brucella* strains *in silico* but failed to isolate further phages. Considering the lengths of most of the prophage sequences it is likely that they do not encode all proteins required for the assembly of intact particles. The *Marinomonas* phage P12026 e.g., possesses a genome of 31.7 kb (Kang et al., 2012) but homologous sequences in *Brucella* have only a length of up to 13.7 kb (Table 1). It can of course not be ruled out that in some cases we did not use suitable indicator strains for the detection of lytic activity. This particularly pertains to a rather conserved prophage that has been detected in *B. suis* 1330 and many other brucellae. It is conceivable that the prophage repressor gene of this phage prevents cell lysis by closely related phages. On the other hand, it should be emphasized that the used computer program (Zhou et al., 2011) did not detect the BiPBO1 prophage in its natural host. The reason for this failure might be that only partial sequences of *B*. *inopinata* BO1 could be analyzed. However, some prophages might be more critical than others to be identified by *in silico* studies.

In spite of the fact that BiPBO1 was exclusively detected in the *B. inopinata* strain BO1, the phage exhibited a rather wide host range within the genus *Brucella*. Moreover, lysogenization of *B. abortus* S19 was simply achieved and the prophage revealed a high stability in this strain and also in its original host. As the BiPBO1 attachment site is present in many brucellae and in other members of the rhizobiales, lysogenization of other species should be possible. Hence, the question arises why this phage was not found in more strains. The answer to this question could be the different habitats of brucellae. *B. inopinata* was isolated from a human breast implant infection and represents the most distant *Brucella* species at the phenotypic and phylogenetic level while it is closely related to *Ochrobactrum* (Scholz et al., 2010; Jimenéz de Bagüés et al., 2014). The animal or environmental reservoir of *B. inopinata* is not known but interestingly a closely related prophage exists in *O. anthropi* ATCC 49188 (Figure 4). We also analyzed this strain and found that it harbors six putative prophages. Upon induction with mitomycin C, phage particles exhibiting different morphologies (myoviral and siphoviral) were released from the cells (data not shown). Furthermore, the BiPBO1-related phage was isolated and characterized. Like BiPB01, this phage is a siphovirus, which, however, lysed several *Ochrobactrum* species, but not *Brucella*. Thus, these closely related phages presumably possess different receptor binding proteins determining host specificity. Nevertheless, the data suggest that at least in the past, mobile genetic elements were exchanged between these species and that phages released from *B. inopinata* did yet obviously not encounter other suitable *Brucella* hosts. Atypical *Brucella* species like *B. inopinata* may therefore represent a link between classical *Brucella* species and *Ochrobactrum* and other rhizobiales (Wattam et al., 2014). Phages released from these environmental bacteria might enter classical *Brucella* strains by an intermediate propagation in atypical brucellae. On the other hand, the 1.5 kb DNA region in many *Brucella* strains that revealed strong homologies to parts of the BiPB01 ORFs 29 and 30 and that is located adjacent to the attachment site B might be a remnant of a former prophage, which was deleted in the course of genome reduction during the evolution from a soil bacterium to a pathogen.

The BiPBO1 genome has a size of 46.9 kb which lies within the common size range of temperate phages. Most BiPBO1 gene products did not disclose similarities to proteins of other phages. Homologies were rather found to hypothetical proteins encoded by predicted prophages residing in various genera, notably *Ochrobactrum*. Nevertheless, some presumably essential genes have been identified. According to the determined data the BiPBO1 genome is similarly organized like the genomes of many other temperate phages. Genes important for virion assembly, integration, replication and host cell lysis are more or less clustered. Though, due to the large number of genes with unknown functions, this impression might change when more information is available on the phage proteins with yet unknown function. Similarly, it can currently not be excluded that BiPBO1 contains genes that cause a lysogenic conversion of the host. Integration of the phage into the bacterial chromosome was not observed within the coding sequence of a gene and we did not detect any metabolic changes in the lysogenized *B. abortus* strain S19. However, other phenotypic properties of the bacteria, e.g., the ability to invade and replicate within cells might be affected. This has to be investigated by further comparison of lysogenic and non-lysogenic strains.

In conclusion, this study showed that temperate phages exist in *Brucella.* To what extent they are involved in horizontal gene transfer has still to be clarified. Nevertheless, the close relationship of *B. inopinata* phage BiPBO1 to an *O. anthropi* phage suggests that gene exchange may occur between these genera. Unfortunately, no information is currently available on temperate phages of *Ochrobactrum.* Our analysis of *O. anthropi* ATCC 49188 shows that they occur in this genus. Hence, it makes sense to study more phages of *Brucella* and *Ochrobactrum* to gain insight into the role that they play for these bacteria.

## Author contributions

JH, SH, KN, and SA designed the study. JH, CG, and JR performed the experiments. JH, CG, JR, KN, SA analyzed the data. All authors prepared the tables and figures, wrote and edited the manuscript.

### Conflict of interest statement

The authors declare that the research was conducted in the absence of any commercial or financial relationships that could be construed as a potential conflict of interest.
