# Supplementary material for: Analysis of the First Temperate Broad Host Range Brucellaphage (BiPBO1) Isolated from B. inopinata
Source: Front Microbiol. 2016 Jan 28;7:24. doi: 10.3389/fmicb.2016.00024 (PMC4729917; doi:10.3389/fmicb.2016.00024)
Supplement: Supplementary file 1 [file Table1.DOCX]

Supplementary Material

**Analysis of the first temperate broad host range brucellaphage (BiPBO1) isolated from *B. inopinata***

**Jens A. Hammerl^*^, Cornelia Göllner, Sascha Al Dahouk, Karsten Nöckler, Jochen Reetz, and Stefan Hertwig**

*** Correspondence:** Corresponding Author: [jens-andre.hammerl@bfr.bund.de](mailto:jens-andre.hammerl@bfr.bund.de)

# Supplementary Tables

**Table S1. Bacterial strains used in this study**

| ***Brucella* spp.** | **Strain** | **Origin** | **Reference collection** |
| --- | --- | --- | --- |
| *B. abortus* bv1 | S19 | USDA, USA | NCTC 8038 |
| *B. abortus* bv1 | 544 | VLA, UK | NCTC 10093; ATCC 23448 |
| *B. abortus* bv2 | 86/8/59 | VLA, UK | NCTC 10501; ATCC 23449 |
| *B. abortus* bv3 | Tulya | VLA, UK | NCTC 10502; ATCC 23450 |
| *B. abortus* bv4 | 292 | VLA, UK | NCTC 10503; ATCC 23451 |
| *B. abortus* bv5 | B3196 | VLA, UK | NCTC 10504; ATCC 23452 |
| *B. abortus* bv6 | 870 | VLA, UK | NCTC 10505; ATCC 23453 |
| *B. abortus* bv7 | 63/75 | VLA, UK | NCTC 10506; ATCC 23454 |
| *B. abortus* bv9 | C68 | VLA, UK | NCTC 10507; ATCC 23455 |
| *B. melitensis* bv1 | 16M | VLA, UK | NCTC 10094; ATCC 23456 |
| *B. melitensis* bv2 | 63/9 | VLA, UK | NCTC 10094; ATCC 23456 |
| *B. melitensis* bv3 | Ether | VLA, UK | NCTC 10509; ATCC 23458 |
| *B. suis* bv1 | 1330 | VLA, UK | NCTC 10316; ATCC 23444 |
| *B. suis* bv2 | Thomsen | VLA, UK | NCTC 10510; ATCC 23445 |
| *B. suis* bv3 | 686 | VLA, UK | NCTC 10511; ATCC 23446 |
| *B. suis* bv4 | 40 | VLA, UK | NCTC 11364; ATCC 23447 |
| *B. suis* bv5 | 513 | VLA, UK | NCTC 11996 |
| *B. ovis* | 63/290 | VLA, UK | NCTC 10512; ATCC 25840 |
| *B. neotomae* | 5K33 | VLA, UK | NCTC 10084; ATCC 23459 |
| *B. canis* | RM6/66 | VLA, UK | NCTC 10854; ATCC 23365 |
| *B. ceti* | B1/94 | VLA, UK | NCTC 12891; BCCN 94-74 |
| *B. pinnipedialis* | B2/94 | VLA, UK | NCTC 12890; BCCN 94-73 |
| *B. microti* | CCM 4915 | VLA, UK | CAPM 6434; BCCN 07-01 |
| *B. inopinata* | BO1 | VLA, UK | CAPM 6436 |
| *B. inopinata*-like | BO2 | VLA, UK | n.a. (Tiller *et al.*, 2010) |
| *Brucella* sp. | F60 | VLA, UK | n.a. (Hofer *et al.*, 2012) |
| *Brucella* sp. | F965 | VLA, UK | n.a. (Hofer *et al.*, 2012) |

**References**

Hofer, E., Revilla-Fernández, S., Al Dahouk, S., Riehm, J.M., Nöckler, K., Zygmunt, M.S., *et al.* (2012). A potential novel *Brucella* species isolated from mandibular lymph nodes of red foxes in Austria. Vet. Microbiol. 155: 93-99.

Tiller, R.V., Gee, J.E., Lonsway, D.R., Gribble, S., Bell, S.C., Jennison, A.V., *et al.* (2010). Identification of an unusual *Brucella* strain (BO2) from a lung biopsy in a 52 year-old patient with chronic destructive pneumonia. BMC Microbiol. 10: 23.
