# Supplementary material for: Analysis of the First Temperate Broad Host Range Brucellaphage (BiPBO1) Isolated from B. inopinata
Source: Front Microbiol. 2016 Jan 28;7:24. doi: 10.3389/fmicb.2016.00024 (PMC4729917; doi:10.3389/fmicb.2016.00024)
Supplement: Supplementary file 2 [file Table2.DOCX]

Supplementary Material

**Analysis of the first temperate broad host range brucellaphage (BiPBO1) isolated from *B. inopinata***

**Jens A. Hammerl^*^, Cornelia Göllner, Sascha Al Dahouk, Karsten Nöckler, Jochen Reetz, and Stefan Hertwig**

*** Correspondence:** Corresponding Author: [jens-andre.hammerl@bfr.bund.de](mailto:jens-andre.hammerl@bfr.bund.de)

# Supplementary Tables

**Table S2. Bacteriophages used in this study**

| **Brucellaphages** | **Description** | **Origin (Reference)** |
| --- | --- | --- |
| F1 | Virulent typing phage | VLA, UK (Hammerl *et al.*, 2014) |
| F25 | Virulent typing phage | VLA, UK (Al Dahouk *et al.*, 2012) |
| BiPBO1 | Temperate phage | *B. inopinata* strain BO1  (This work) |

**References**

Al Dahouk, S., Hofer, E., Tomaso, H., Vergnaud, G., Le Flèche, P., Cloeckaert, A. *et al.* (2012). Intraspecies biodiversity of the genetically homologous species *Brucella microti*. Appl. Environ. Microbiol. 78(5):1534-1543.

Hammerl, J.A., Al Dahouk, S., Nöckler, K., Göllner, C., Appel, B., Hertwig, S. (2014). F1 and tbilisi are closely related brucellaphages exhibiting some distinct nucleotide variations which determine the host specificity. Genome Announc. 2(1): e01250-13.
