# Supplementary material for: Analysis of the First Temperate Broad Host Range Brucellaphage (BiPBO1) Isolated from B. inopinata
Source: Front Microbiol. 2016 Jan 28;7:24. doi: 10.3389/fmicb.2016.00024 (PMC4729917; doi:10.3389/fmicb.2016.00024)
Supplement: Supplementary file 3 [file Table3.DOCX]

Supplementary Material

**Analysis of the first temperate broad host range brucellaphage (BiPBO1) isolated from *B. inopinata***

**Jens A. Hammerl^*^, Cornelia Göllner, Sascha Al Dahouk, Karsten Nöckler, Jochen Reetz, and Stefan Hertwig**

*** Correspondence:** Corresponding Author: [jens-andre.hammerl@bfr.bund.de](mailto:jens-andre.hammerl@bfr.bund.de)

# Supplementary Tables

**Table S3. ORF analysis of the BiPBO1 genome**

| **ORF No.** | **Start** | **Stop** | **Strand** | **Predicted function** | **Best match** | **E-value** | **Amino acid identities** | **Accession no.** |
| --- | --- | --- | --- | --- | --- | --- | --- | --- |
| ORF01 | 1 | 402 | + | Phage terminase, small subunit | Hypothetical protein [Ochrobactrum anthropi] | 1.00E-86 | 128/133 (96%) | WP_036586388.1 |
| ORF02 | 399 | 2,087 | + | Phage terminase, large subunit | Hypothetical protein [Ochrobactrum anthropi] | 0.0 | 536/555 (97%) | WP_036586386.1 |
| ORF03 | 2,087 | 3,319 | + | Phage portal protein | Hypothetical protein IL59_0212130 [Brucella suis bv. 4 str. 40] | 0.0 | 410/410 (100%) | KEY04143.1 |
| ORF04 | 3,297 | 3,497 | + | Hypothetical protein | No significant homologies | - | - | - |
| ORF05 | 3,475 | 4,320 | + | Phage Clp protease (peptidase) | Hypothetical protein [Oceanicola nanhaiensis] | 4.00E-151 | 206/270 (76%) | WP_028286089.1 |
| ORF06 | 4,334 | 5,626 | + | Phage major capsid protein | Phage capsid protein [Paracoccus aminophilus] | 0.0 | 254/431 (59%) | WP_020949982.1 |
| ORF07 | 5,690 | 5,851 | + | Hypothetical protein (phage) | Hypothetical protein [Kiloniella sp. P1-1] | 3.00E-11 | 27/40 (68%) | WP_046509871.1 |
| ORF08 | 5,855 | 6,436 | + | Phage DNA packaging/ head-tail-connector | Hypothetical protein [Sinorhizobium fredii] | 1.00E-48 | 193/193 (100%) | WP_037437203.1 |
| ORF09 | 6,436 | 6,789 | + | Phage head-tail-adapter | Phage head-tail adapter protein [Ochrobactrum anthropi] | 3.00E-70 | 107/117 (91%) | WP_011982436.1 |
| ORF10 | 6,794 | 7,228 | + | Phage head/tail component | HK97 family phage protein [Ochrobactrum rhizosphaerae] | 4.00E-75 | 107/144 (74%) | WP_024900311.1 |
| ORF11 | 7,225 | 7,626 | + | Hypothetical protein (phage) | hypothetical protein [Ochrobactrum anthropi] | 3.00E-81 | 117/132 (89%) | WP_011982438.1 |
| ORF12 | 7,623 | 7,829 | + | Hypothetical protein (phage) | Hypothetical protein [Ochrobactrum anthropi] | 9.00E-40 | 64/67 (96%) | WP_041544904.1 |
| ORF13 | 7,908 | 8,360 | + | Phage major tail protein | Hypothetical protein [Ochrobactrum anthropi] | 5.00E-95 | 129/147 (88%) | WP_011982439.1 |
| ORF14 | 8,360 | 8,761 | + | Hypothetical protein (phage) | Hypothetical protein [Ochrobactrum sp. UNC390CL2Tsu3S39] | 5.00E-84 | 124/133 (93%) | WP_036596028.1 |
| ORF15 | 8,782 | 8,904 | + | hypothetical protein | p016 [Rhizobium phage 16-3] | 4.00E-07 | 21/40 (53%) | YP_002117575.1 |
| ORF16 | 8,904 | 11,489 | + | Phage tail length tape measure protein | Hypothetical protein [Ochrobactrum sp. UNC390CL2Tsu3S39] | 0.0 | 641/703 (91%) | WP_029926888.1 |
| ORF17 | 11,489 | 12,169 | + | Hypothetical protein | Hypothetical protein [Ochrobactrum anthropi] | 3.00E-157 | 220/224 (98%) | WP_011982442.1 |
| ORF18 | 12,169 | 12,834 | + | Transcription regulator? | Hypothetical protein Oant_0253 [Ochrobactrum anthropi ATCC 49188] | 4.00E-152 | 208/214 (97%) | ABS12984.1 |
| ORF19 | 13,158 | 12,862 | - | Addiction module antitoxin/toxin | Transcriptional regulator [Ochrobactrum] | 2.00E-53 | 87/99 (88%) | WP_006470801.1 |
| ORF20 | 13,450 | 13,160 | - | Addiction module antitoxin/toxin | Hypothetical protein [Ochrobactrum] | 4.00E-59 | 90/96 (94%) | WP_021586313.1 |
| ORF21 | 13,946 | 13,560 | - | Hypothetical protein | Hypothetical protein [Ochrobactrum sp. UNC390CL2Tsu3S39] | 4.00E-30 | 59/133 (44%) | WP_029924509.1 |
| ORF22 | 14,049 | 14,243 | + | Hypothetical protein | Hypothetical protein [Acetobacteraceae bacterium AT-5844] | 4.00E-07 | 27/53 (51%) | WP_040291466.1 |
| ORF23 | 14,358 | 15,101 | + | Phage antirepressor (KilA) | Hypothetical protein IL59_0215650 [Brucella suis bv. 4 str. 40] | 0.0 | 247/247 (100%) | KEY03590.1 |
| ORF24 | 15,101 | 15,358 | + | Hypothetical protein | Hypothetical protein [Chelativorans sp. J32] | 8.00E-10 | 27/50 (54%) | WP_028034124.1 |
| ORF25 | 15,358 | 15,594 | + | Hypothetical protein | Hypothetical protein [Ochrobactrum anthropi] | 5.00E-37 | 65/78 (83%) | WP_036586364.1 |
| ORF26 | 15,604 | 15,903 | + | Hypothetical protein | Hypothetical protein [Ochrobactrum anthropi] | 2.00E-48 | 77/84 (92%) | WP_036586362.1 |
| ORF27 | 16,324 | 15,995 | - | Hypothetical protein | Hypothetical protein [Ensifer sp. Br816] | 2.00E-22 | 48/94 (51%) | WP_018240560.1 |
| ORF28 | 16,375 | 16,767 | + | Hypothetical protein | Hypothetical protein [Ochrobactrum sp. UNC390CL2Tsu3S39] | 1.00E-79 | 103/128 (80%) | WP_029926895.1 |
| ORF29 | 16,758 | 18,530 | + | Fibronectin | Fibronectin [Ochrobactrum anthropi] | 0.0 | 562/590 (95%) | WP_011982446.1 |
| ORF30 | 18,596 | 20,461 | + | Pectate lyase superfamily protein | Hypothetical protein IL59_0214975 [Brucella suis bv. 4 str. 40] | 0.0 | 398/398 (100%) | KEY03708.1 |
| ORF31 | 20,475 | 20,705 | + | Hypothetical protein | No significant homologies | - | - | - |
| ORF32 | 20,853 | 22,001 | + | Acyltransferase | Hypothetical protein [Mesorhizobium] | 2.00E-68 | 156/364 (43%) | WP_023773702.1 |
| ORF33 | 22,107 | 22,790 | + | Phage lysozyme | Hypothetical protein [Rhizobium sp. LMB-1] | 2.00E-114 | 166/227 (73%) | WP_045534055.1 |
| ORF34 | 22,787 | 23,062 | + | Hypothetical protein | Hypothetical protein [Brucella sp. BO2] | 3.00E-34 | 85/91 (93%) | WP_042971836.1 |
| ORF35 | 23,174 | 23,479 | + | Hypothetical protein | Hypothetical protein [Brucella sp. BO2] | 2.00E-63 | 100/101 (99%) | WP_025199134.1 |
| ORF36 | 23,677 | 23,483 | - | Hypothetical protein | Hypothetical protein [Ochrobactrum sp. UNC390CL2Tsu3S39] | 3.00E-34 | 62/64 (97%) | WP_029924588.1 |
| ORF37 | 24,990 | 23,947 | - | Phage integrase | Phage integrase family [Shinella sp. DD12] | 4.00E-138 | 200/351 (57%) | WP_024270545.1 |
| ORF38 | 25,216 | 24,941 | - | Excisionase? | Hypothetical protein [Xanthobacter autotrophicus] | 8.00E-18 | 32/64 (50%) | WP_041577266.1 |
| ORF39 | 25,562 | 25,296 | - | Hypothetical protein | Hypothetical protein Oant_1538 [Ochrobactrum anthropi ATCC 49188] | 1.00E-19 | 45/88 (51%) | ABS14254.1 |
| ORF40 | 25,646 | 25,879 | + | Hypothetical protein | Hypothetical protein [Ochrobactrum sp. UNC390CL2Tsu3S39] | 2.00E-34 | 58/77 (75%) | WP_029924477.1 |
| ORF41 | 26,055 | 25,885 | - | Hypothetical protein | No significant homologies | - | - | - |
| ORF42 | 26,263 | 26,048 | - | Hypothetical protein | No significant homologies | - | - | - |
| ORF43 | 26,487 | 26,260 | - | Hypothetical protein | Hypothetical protein [Brucella sp. BO2] | 4.00E-18 | 49/87 (56%) | WP_025199067.1 |
| ORF44 | 26,653 | 26,477 | - | Hypothetical protein | Hypothetical protein [Ochrobactrum rhizosphaerae] | 2.00E-13 | 32/48 (67%) | WP_024898852.1 |
| ORF45 | 26,871 | 26,650 | - | hypothetical protein | No significant homologies | - | - | - |
| ORF46 | 27,011 | 26,868 | - | hypothetical protein | No significant homologies | - | - | - |
| ORF47 | 27,789 | 27,094 | - | hypothetical protein | Hypothetical protein [Brucella sp. BO2] | 4.00E-41 | 93/189 (49%) | WP_025199072.1 |
| ORF48 | 28,099 | 27,779 | - | RNA-binding protein | Hypothetical protein [Ochrobactrum sp. UNC390CL2Tsu3S39] | 6.00E-37 | 61/103 (59%) | WP_029926786.1 |
| ORF49 | 28,746 | 28,096 | - | Hypothetical protein (phage) | Phage protein [Ochrobactrum sp. UNC390CL2Tsu3S39] | 6.00E-11 | 50/113 (44%) | WP_029926788.1 |
| ORF50 | 29,069 | 28,743 | - | Hypothetical protein | Hypothetical protein [Bordetella bronchiseptica] | 0.0024 | 35/107 (33%) | WP_033447595.1 |
| ORF51 | 29,491 | 29,066 | - | HNH endonuclease | HNH endonuclease [Ochrobactrum anthropi] | 2.00E-69 | 108/130 (83%) | WP_036586439.1 |
| ORF52 | 30,830 | 29,493 | - | Recombinational DNA repair protein RecT | Hypothetical protein [Ochrobactrum anthropi] | 0.0 | 374/442 (85%) | WP_011982403.1 |
| ORF53 | 31,756 | 30,833 | - | 5'-3' specific dsDNA exonuclease (VIII) RecE | Hypothetical protein [Ochrobactrum anthropi] | 0.0 | 298/307 (97%) | WP_011982404.1 |
| ORF54 | 32,030 | 31,749 | - | Transcriptional repressor | Hypothetical protein D584_22836 [Ochrobactrum intermedium M86] | 9.00E-20 | 50/94 (53%) | ELT46872.1 |
| ORF55 | 32,293 | 32,030 | - | Hypothetical protein | Hypothetical protein [Ochrobactrum anthropi] | 4.00E-49 | 75/87 (86%) | WP_036586434.1 |
| ORF56 | 32,471 | 32,295 | - | Hypothetical protein | Hypothetical protein [Ochrobactrum anthropi] | 2.00E-09 | 36/65 (55%) | WP_036586431.1 |
| ORF57 | 33,010 | 32,468 | - | Hypothetical protein | Hypothetical protein BW39_03904 [Delftia sp. RIT313] | 9.00E-52 | 91/171 (53%) | EZP51435.1 |
| ORF58 | 33,137 | 33,015 | - | Hypothetical protein | Hypothetical protein [Ochrobactrum anthropi] | 4.00E-15 | 35/40 (88%) | WP_036586429.1 |
| ORF59 | 33,428 | 33,207 | - | Hypothetical protein | No significant homologies | - | - | - |
| ORF60 | 33,769 | 33,428 | - | Hypothetical protein | Hypothetical protein [Ochrobactrum anthropi] | 1.00E-52 | 84/113 (74%) | WP_043062345.1 |
| ORF61 | 34,156 | 33,782 | - | DNA-binding protein? | Hypothetical protein [Ochrobactrum intermedium] | 6.00E-26 | 47/61 (77%) | WP_025090249.1 |
| ORF62 | 34,397 | 34,263 | - | Hypothetical protein | No significant homologies | - | - | - |
| ORF63 | 34,733 | 34,575 | - | Hypothetical protein | No significant homologies | - | - | - |
| ORF64 | 35,200 | 34,760 | - | Hypothetical protein | Hypothetical protein [Brucella sp. BO2] | 6.00E-100 | 145/146 (99%) | WP_025199079.1 |
| ORF65 | 35,504 | 35,190 | - | Hypothetical protein | Hypothetical protein [Brucella sp. BO2] | 2.00E-69 | 103/104 (99%) | WP_009363438.1 |
| ORF66 | 35,758 | 35,525 | - | hypothetical protein | Hypothetical protein [Ochrobactrum intermedium] | 3.00E-26 | 50/72 (69%) | WP_006470380.1 |
| ORF67 | 36,384 | 35,779 | - | Type VI secretion protein | Type VI secretion system-associated protein [Rhizobium gallicum R602] | 4.00E-45 | 85/212 (40%) | AJD43764.1 |
| ORF68 | 37,196 | 36,393 | - | Prophage repressor | Transcriptional regulator [Brucella suis bv. 4 str. 40] | 0.0 | 259/259 (100%) | KEY04129.1 |
| ORF69 | 37,211 | 37,462 | + | Repressor? | Hypothetical protein [Ochrobactrum intermedium] | 8.00E-45 | 71/75 (95%) | WP_025091483.1 |
| ORF70 | 37,459 | 37,929 | + | Hypothetical protein | Hypothetical protein [Ochrobactrum anthropi] | 2.00E-106 | 150/156 (96%) | WP_036586413.1 |
| ORF71 | 38,042 | 38,293 | + | Hypothetical protein | Hypothetical protein [Brucella sp. BO2] | 7.00E-24 | 52/83 (63%) | WP_025199085.1 |
| ORF72 | 38,537 | 38,259 | - | Hypothetical protein | No significant homologies | - | - | - |
| ORF73 | 38,776 | 38,633 | - | Hypothetical protein | No significant homologies | - | - | - |
| ORF74 | 38,855 | 39,253 | + | Hypothetical protein | Hypothetical protein [Ochrobactrum anthropi] | 9.00E-85 | 127/131 (97%) | WP_011982413.1 |
| ORF75 | 39,207 | 39,332 | + | hypothetical protein | No significant homologies | - | - | - |
| ORF76 | 39,329 | 39,865 | + | hypothetical protein | Hypothetical protein [Brucella sp. BO2] | 8.00E-104 | 178/178 (100%) | WP_025199087.1 |
| ORF77 | 39,865 | 40,614 | + | Chromosomal replication initiator protein DnaA | Hypothetical protein [Brucella sp. BO2] | 3.00E-176 | 244/249 (98%) | WP_025199088.1 |
| ORF78 | 40,713 | 41,402 | + | Hypothetical protein (phage) | Hypothetical protein [Ochrobactrum anthropi] | 1.00E-161 | 220/229 (96%) | WP_011982418.1 |
| ORF79 | 41,417 | 42,901 | + | Replicative DNA helicase | Helicase DnaB [Ochrobactrum anthropi] | 0.0 | 486/494 (98%) | WP_011982419.1 |
| ORF80 | 42,988 | 43,206 | + | Hypothetical protein | Hypothetical protein [Ochrobactrum anthropi] | 1.00E-35 | 63/72 (88%) | WP_041544893.1 |
| ORF81 | 43,203 | 43,706 | + | DNA repair exonuclease | Hypothetical protein [Brucella sp. BO2] | 9.00E-102 | 143/144 (99%) | WP_029669470.1 |
| ORF82 | 43,703 | 44,143 | + | hypothetical protein | Hypothetical protein [Ochrobactrum anthropi] | 1.00E-102 | 144/146 (99%) | WP_043062354.1 |
| ORF83 | 44,140 | 44,922 | + | hypothetical protein | Hypothetical protein [Brucella sp. BO2] | 0.0 | 251/260 (97%) | WP_025199096.1 |
| ORF84 | 45,651 | 45,800 | + | hypothetical protein | No significant homologies | - | - | - |
| ORF85 | 45,797 | 46,066 | + | hypothetical protein | Hypothetical protein [Ochrobactrum anthropi] | 2.00E-19 | 56/81 (69%) | WP_043062360.1 |
| ORF86 | 46,066 | 46,263 | + | hypothetical protein | Hypothetical protein [Brucella sp. BO2] | 1.00E-36 | 60/65 (92%) | WP_042971814.1 |
| ORF87 | 46,685 | 46,518 | - | hypothetical protein | No significant homologies | - | - | - |

Sequence analysis and alignments were carried out using Accelrys Gene v2.5 (Accelrys Inc., San Diego, CA, USA). ORF analyses were performed using the algorithms of MyRAST (Aziz *et al.*, 2008; Glass *et al.*, 2010; Meyer *et al.*, 2008) and ORF Finder (NCBI) (gene product size of >29 aa) (Rombel *et al.*, 2002). Similarity and identity values were determined at the NCBI homepage using standard parameters of different BLAST algorithms (Johnson *et al.*, 2008).

**References**

Aziz, R.K., Bartels, D., Best, A.A., DeJongh, M., Disz, T., Edwards, R.A. *et al.* (2008). The RAST Server: rapid annotations using subsystems technology. BMC Genomics 9:75.

Glass, E.M., Wilkening, J., Wilke, A., Antonopoulos, D., Meyer, F. (2010). Using the metagenomics RAST server (MG-RAST) for analyzing shotgun metagenomes. Cold Spring Harb. Protoc. 2010:db.

Johnson, M., Zaretskaya, I., Raytselis, Y., Merezhuk, Y., McGinnis, S., Madden, T.L. (2008). NCBI BLAST: A better web interface. Nucleic Acids Res. 36:W5-W9.

Meyer, F., Paarmann, D., D'Souza, M., Olson, R., Glass, E.M., Kubal, M. *et al.* (2008). The metagenomics RAST server - a public resource for the automatic phylogenetic and functional analysis of metagenomes. BMC Bioinformatics 9:386.

Rombel, I.T., Sykes, K.F., Rayner, S., Johnston, S.A. (2002). ORF-FINDER: A vector for high-throughput gene identification. Gene 282:33-41.
