# Supplementary material for: Analysis of the First Temperate Broad Host Range Brucellaphage (BiPBO1) Isolated from B. inopinata
Source: Front Microbiol. 2016 Jan 28;7:24. doi: 10.3389/fmicb.2016.00024 (PMC4729917; doi:10.3389/fmicb.2016.00024)
Supplement: Supplementary file 4 [file Table4.DOCX]

Supplementary Material

**Analysis of the first temperate broad host range brucellaphage (BiPBO1) isolated from *B. inopinata***

**Jens A. Hammerl^*^, Cornelia Göllner, Sascha Al Dahouk, Karsten Nöckler, Jochen Reetz, and Stefan Hertwig**

*** Correspondence:** Corresponding Author: [jens-andre.hammerl@bfr.bund.de](mailto:jens-andre.hammerl@bfr.bund.de)

# Supplementary Tables

**Table S4. Identification of Rho–independent transcription terminators using ARNOLD software.**

| **Trans-cription terminator** | **Binding position** | **Motif** | **Strand** | **Sequence (5‘-3‘)** | **Energy (kcal/mol)** |
| --- | --- | --- | --- | --- | --- |
| TT01 | 2 336 | Rnamotif | + | AAAGAGAAGGCCCGTGAAAGCGGTCTTTTTTCATT | -6.30 |
| TT02 | 7 474 | Rnamotif | - | TCTGGAGTATCGCGCTGAAGCGCTTTTTTGACGCC | -6.80 |
| TT03 | 7 837 | Rnamotif | + | CACCTGAAACCGGGCCTCAAGCCCTTTTAAGGCTGG | -6.90 |
| TT04 | 7 856 | Rnamotif | + | AGCCCTTTTAAGGCTGGCGAATAGCTGGCCcgTTTTCGCATGGA | -11.60 |
| TT05 | 10 529 | Rnamotif | - | CCAGAGCCACCGCCAAATCCAGTCGTGTTTGGCTTGTTGTTTGGC | -7.90 |
| TT06 | 12 829 | Rnamotif | - | GCCTGACGCTGCCCCGCCACGGCGGGGTTTTTGCTAACC | -13.80 |
| TT07 | 14 288 | Both | - | GATTACGCCGACGCGCTAACGTCAGCGTGgTTTTTCCATCGG | -8.50 |
| TT08 | 15 963 | Both | + | CGCTAATGGAGAGCGCCCTACGGGGCGCTTTTCTATTTGCAA | -16.60 |
| TT09 | 18 062 | Rnamotif | + | CATCCAATACCCGCTGTTTGGCATCAACAGTGTTTTTGAAGTCC | -6.30 |
| TT10 | 18 547 | Rnamotif | + | AGAAACCCACACCCTGCCTTGGCGGGGcgTTTTGCTATGGA | -11.70 |
| TT11 | 22 050 | Both | + | CTAATTTCCCAGGCCCTGCATTTGCGGGGCCTTTTCTTTTGCC | -17.80 |
| TT12 | 22 817 | Rnamotif | + | AAATACTCGCTCGCTGCCCTTGTGGCGGCGTTTTTGTTGGCA | -11.90 |
| TT13 | 34 135 | Both | - | ATCCCACATGTCATGCAACATGTTGCGTGTTATTTTTTTGT | -7.90 |
| TT14 | 34 178 | Erpin | - | GACCGCTTTAGGCCCCGGCAGCATCACCGCTCCGGGGCTTTTCTTTTATTC | -13.92 |
| TT15 | 34 725 | Both | - | AGTAACACCAGCCCCGCTTCGGCGGGGTTTTTTGTTTGCG | -15.70 |
| TT16 | 37 216 | Rnamotif | + | TGGTGTTGATATTGTCGACATGAAACACGTTGACGAcaTTTTCCAGACAT | -7.60 |
| TT17 | 38 542 | Rnamotif | + | ATCGGTTCCTTCTCGGATTTGGTATTTCGAGTTTTGATACCTC | -5.90 |
| TT18 | 39 695 | Rnamotif | - | CGGGTGTAATCCTGCCCATCCTGGGTAGcTTCGTTGTACGG | -8.80 |
| TT19 | 45 453 | Rnamotif | + | GCGCTATTTCAGCCGTCGCCTTCTGGTGGCGGCTTTTCCGCATCA | -15.90 |

For terminators located on the minus strand, plus strand coordinates are given. Blue and red nucleotides indicate terminator stems and loops, respectively. Lowercase letters of RNA motif predictions indicate spacer elements between the stem-loop and T-rich regions.
