# Supplementary material for: Analysis of the First Temperate Broad Host Range Brucellaphage (BiPBO1) Isolated from B. inopinata
Source: Front Microbiol. 2016 Jan 28;7:24. doi: 10.3389/fmicb.2016.00024 (PMC4729917; doi:10.3389/fmicb.2016.00024)
Supplement: Supplementary file 5 [file Table5.DOCX]

Supplementary Material

**Analysis of the first temperate broad host range brucellaphage (BiPBO1) isolated from *B. inopinata***

**Jens A. Hammerl^*^, Cornelia Göllner, Sascha Al Dahouk, Karsten Nöckler, Jochen Reetz, and Stefan Hertwig**

*** Correspondence:** Corresponding Author: [jens-andre.hammerl@bfr.bund.de](mailto:jens-andre.hammerl@bfr.bund.de)

# Supplementary Tables

**Table S5. Primers used in this study**

| **Primer** | **Sequence 5‘-3‘** | **Target** | **Application** |
| --- | --- | --- | --- |
| BO1capF | ATGTACCCAGCAAGGAAGGC | BiPBO1 major capsid protein gene | Detection of BiPBO1-like phages, product size: 774 bp |
| BO1capR | GCGGCTGCCACAGATAATTG |  |  |
| BO1intF | AGTCAGACAAGACCCGCAAG | BiPBO1 integrase gene | Detection of BiPBO1-like phages, product size: 413 bp |
| BO1intR | AAGTGTCTTGCCGTCGTAGG |  |  |
| BO1repF | TAGGTTATCGCTTTCGGCCC | BiPBO1 replicase gene | Detection of BiPBO1-like phages, product size: 609 bp |
| BO1refR | ACCGCGGCTTTTTGGATAGA |  |  |
| BO1int-outF | TGAGAGGCACAGCAGTAACG | BiPBO1 integrase gene | Determination of the BiPB01 chromosomal integration site (outward PCR) in *B. inopinata* BO1 |
| BO1int-outR | CGGGTCTTGTCTGACTTGCT | BiPBO1 integrase gene |  |
| BPBO1 intoutF | GGAAAAATTGAGCTTGCAGAG | BiPBO1 | Multiplex-PCR for the analysis of the BiPB01 integration site in *Brucella* strains |
| BPBO1 shockinR | TGTGATGCTTGCCCATCTC | BiPBO1 |  |
| BPBO1 S19 shockinR | TGTGGTGCTTGCCCATCTC | BiPBO1 |  |
| S19lysBO1-F | CCATTCCATCCTCAAATACCG | *Brucella* chromosome |  |
| S19lysBO1-R | GTTTATCCGTATTCGCATTGC | *Brucella* chromosome |  |
| S19 phainF | AGCGCATATGATCGCCATCC | *Brucella* chromosome |  |
